# Ability to gain religious experiences as a part of cognitive abilities

**Published:** 2014-07-04

**Authors:** Abdorreza Naser Moghadasi

**Affiliations:** Department of Neurology, School of Medicine, MS Research Center, Neuroscience Institute, Sina Hospital, Tehran University of Medical Science, Tehran, Iran

**Keywords:** Neurotheology, Cognition, Religious Experience

I eagerly studied Sayadmansour’s article.^1^ To my knowledge, this is the first study introducing neurotheology to Iranian readers. However, the article is more than just an introduction and many interesting points can be found among the lines; a particularly remarkable point was including some elements like numbers and better performance of brain through following a series of mathematical systems that are also presented in Islam and Shi’a. The present article can be an opening for a new study due to the fact that most neurotheology studies have been conducted on faithful followers of Christianity and Buddhism and new studies on Muslims can open a new window into the field.

The author correctly refers to the necessity of establishing a relationship between neuroscience and theology and the point that this can lead to achieving new insights in the field of neuroscience and the manner of human exposure to the surrounding world as well as promoting theological perceptions; however, I believe that the article does not determine the position of neurotheology. We cannot figure out the limits of neurotheology and more importantly, unfair expectations from this newfound science after studying the article. In my opinion, only a historical outlook to neurotheology can answer this question.

Perhaps one of the primary objectives of neurotheology was demoting religious and mystic experiences to neurophysiologic activities. God Helmet made by Michael Persinger^2^ was a true picture of the same belief. It was actually an apparatus through which, the temporal lobe was exposed to a weak magnetic field. Persinger has reported that many of the examinees had a “sensed presence”.^2^ He concluded that most mystical experiences are associated with the temporal lobe, and they can occur merely by stimulating the temporal role without the presence of a religious object. Although Persinger’s work was criticized, searching for a God spot continued. Newberg et al.^3^has also conducted remarkable studies on the same field. Following a research on Buddhist monks using single-photon emission computed tomography, Newberg cited that feeling of integrity with the world seen in different mystical schools results from the reduction in the parietal lobe activity.^3^ Studies brought hope for scientists to define a neurophysiologic framework for mystical experiences. Yet, studies of Beauregard carried out using functional magnetic resonance imaging showed that mystical and religious experiences, like many other higher cortical functions, are complicated, and several spots are involved.^4^ In other words, there is no God spot. This might seem to be a hasty assumption, but the complexity of religious experiences imply that such experiences might be a part of human cognitive abilities that grows; at the same time, with others such as language, dream, and reasoning; that is, the human brain is planned to acquire religious experiences through evolution and natural selection. The process does not suggest that religious experiences are originated from our cognitive power; instead, it just shows that our brain is quite ready for acquiring such experiences.

## References

[1] Sayadmansour A (2014). Neurotheology: The relationship between brain and religion. Iran J Neurol.

[2] Ruttan LA, Persinger MA, Koren S (1990). Enhancement of Temporal Lobe-Related Experiences During Brief Exposures to Milligauss Intensity Extremely Low Frequency Magnetic Fields. Journal of Bioelectricity.

[3] Newberg A1, Alavi A, Baime M, Pourdehnad M, Santanna J, d'Aquili E (2001). The measurement of regional cerebral blood flow during the complex cognitive task of meditation: a preliminary SPECT study. Psychiatry Res.

[4] Mario Beauregard M, Denyse O’Leary D (2007). The Spiritual Brain: A Neuroscientist’s Case for the Existence of the Soul.

[5] Newberg A, D'Aquili EG (2008). Why God Won't Go Away: Brain Science and the Biology of Belief.

