# Supplementary material for: Ability to gain religious experiences as a part of cognitive abilities
Source: Iran J Neurol. 2014 Jul 4;13(3):191–2. (PMC4240941)
Supplement: Related texts

# A short response to “Ability to gain religious experiences as a part of cognitive abilities”

**Published:** 2014-07-04

**Authors:** Alireza Sayadmansour

**Affiliations:** Department of Philosophy, School of Literature and Humanities, University of Tehran, Tehran, Iran

First of all, I would wholeheartedly like to thank the sender of the letter for his/her concentration upon my most recent publication. The letter has definitely been written with an open eye on current neurotheological literature. There are a few illustrating point to raise though (my essential words are italicized).

As neurotheology is an emerging (newly born) field of study, both in terms of content and applicability, it sounds quite difficult to ascertain the field’s position among many more. Accordingly, to determine the boundaries of neurotheology is to be a matter of understanding its *multidisciplinarity* drawing attention to the intersection of the brain and religion from at least seven divergent fields. Having pursued current neurotheological debates, one can find out the fact that the sender is right-minded about maintaining that “only a historical outlook” (which is *future-orientedly progressive*) can supply curious minds with an appropriate response to the question whether or not we can disclose “unfair expectations from this newfound science”.

As for the colliding utterance which reminds us about “perhaps one of the primary objectives of neurotheology” as such, is to *degrade* spiritual experiences to “neurophysiologic activities”, I should warn the readers against such a reductionistic standpoint! To me, future neurotheological scholarship must seek for a potentially better evaluation of the specifics of distinctive brain processes so as to determine if and how they belong to religious concepts in general. Generally speaking, there are two main possible attitudes toward neurotheology: the first attitude throws light of inquiry upon neurothological issues from a theo-spiritual perspective according to which a real religious entity compels the brain to generate religious experiences; therefore, the arrow of neurotheology extends from religion to the brain causally. Conversely, the second one considers the arrow as extending from the brain (more empirically seen) to religion. In other words, many neuroscientists tend to treat theo-spiritual experiences as originating from neurophysiologic processes. That is why Newberg and D'Aquili speak enthusiastically of “photographs of God”.^5^ Ultimately, I encourage readers to have both arrow-directions in mind when conducting research on neurotheology.

## Notes

**How to cite this article: **Naser Moghadasi A. Ability to gain religious experiences as a part of cognitive abilities. Iran J Neurol 2014; 13(3): 191-2.

## References

[1] Sayadmansour A (2014). Neurotheology: The relationship between brain and religion. Iran J Neurol.

[2] Ruttan LA, Persinger MA, Koren S (1990). Enhancement of Temporal Lobe-Related Experiences During Brief Exposures to Milligauss Intensity Extremely Low Frequency Magnetic Fields. Journal of Bioelectricity.

[3] Newberg A1, Alavi A, Baime M, Pourdehnad M, Santanna J, d'Aquili E (2001). The measurement of regional cerebral blood flow during the complex cognitive task of meditation: a preliminary SPECT study. Psychiatry Res.

[4] Mario Beauregard M, Denyse O’Leary D (2007). The Spiritual Brain: A Neuroscientist’s Case for the Existence of the Soul.

[5] Newberg A, D'Aquili EG (2008). Why God Won't Go Away: Brain Science and the Biology of Belief.
